# Characterization of meat quality traits, fatty acids and volatile compounds in Hu and Tan sheep

**DOI:** 10.3389/fnut.2023.1072159

**Published:** 2023-02-14

**Authors:** Jing Li, Chaohua Tang, Youyou Yang, Ying Hu, Qingyu Zhao, Qing Ma, Xiangpeng Yue, Fadi Li, Junmin Zhang

**Affiliations:** ^1^State Key Laboratory of Animal Nutrition, Institute of Animal Sciences of Chinese Academy of Agricultural Sciences, Beijing, China; ^2^Scientific Observing and Experiment Station of Animal Genetic Resources and Nutrition in North China of Ministry of Agriculture and Rural Affairs, Institute of Animal Sciences of Chinese Academy of Agricultural Sciences, Beijing, China; ^3^Institute of Animal Science, Ningxia Academy of Agricultural and Forestry Sciences, Yinchuan, China; ^4^State Key Laboratory of Grassland Agro-ecosystems; Key Laboratory of Grassland Livestock Industry Innovation, Ministry of Agriculture and Rural Affairs; Engineering Research Center of Grassland Industry, Ministry of Education; College of Pastoral Agriculture Science and Technology, Lanzhou University, Lanzhou, China

**Keywords:** breed, intramuscular fat, lamb quality, odorant, odor activity value, volatilomics

## Abstract

Sheep breed has a major influence on characteristics of meat quality and intramuscular fat (IMF), however, studies into the relationship between sheep breed and meat quality traits rarely consider the large variation in IMF within breed. In this study, groups of 176 Hu and 76 Tan male sheep were established, weaned at 56 days old, with similar weights, and representative samples were selected based on the distribution of IMF in each population, to investigate variations in meat quality, IMF and volatile compound profiles between breeds. Significant differences were observed in drip loss, shear force, cooking loss, and color coordinates between Hu and Tan sheep (*p* < 0.01). The IMF content and the predominate unsaturated fatty acids, oleic and *cis*, *cis*-linoleic acids, were similar. Eighteen out of 53 volatile compounds were identified as important odor contributors. Of these 18 odor-active volatile compounds, no significant concentration differences were detected between breeds. In another 35 volatile compounds, γ-nonalactone was lower in Tan sheep relative to Hu sheep (*p* < 0.05). In summary, Tan sheep exhibited lower drip loss, higher shear force values, and redder color, had less saturated fatty acids, and contained less γ-nonalactone against Hu sheep. These findings improve understanding of aroma differences between Hu and Tan sheep meat.

**Graphical Abstract:**
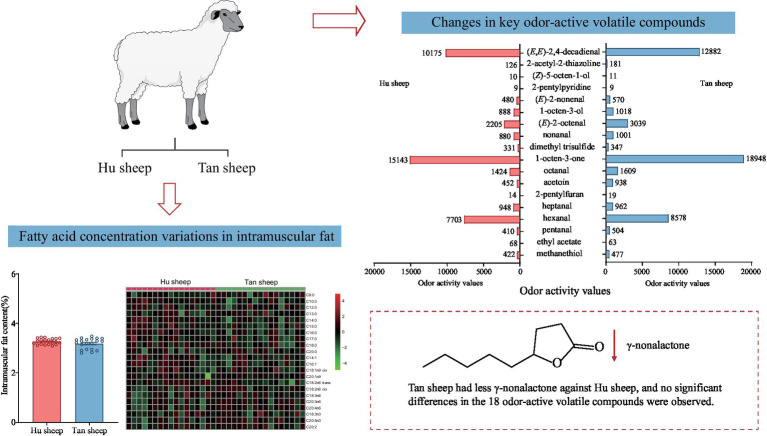

## Highlights

– Populations were established with 176 Hu and 76 Tan male sheep.
– Intra-breed variation in intramuscular fat content in both breeds was large.
– Intramuscular fat content was similar between breeds.
– Tan sheep fat had less γ-nonalactone than Hu sheep.

## 1. Introduction

Lamb eating quality is critical to consumer acceptance and repurchase decisions. Sheep breed (genotype) is a very important factor influencing meat quality and many meat quality traits appear to be heritable, so genetic breeding strategies have potential to improve meat quality (1, 2). Sheep germplasm resources in China are rich and a great resource for genetic breeding. Of these, Tan sheep breed is in high demand from consumers, due to its unique flavor and texture (3). The production of lamb from Tan sheep has been consistently rising in Ningxia Hui Autonomous Region, the dominant production area, at an annual average rate of 27.32% from 2015 to 2021. Hu sheep with the top market share in China is the predominant sheep breed for overall lamb production due to early maturity, multiple lambs per litter, and year-round estrus. Recent research has focused on investigating differences in meat quality between Hu and Tan sheep, the main genes related to meat quality and their mechanisms of action (4–6). However, information on differences in eating quality, especially volatile compound profiles, between Hu and Tan sheep is not available.

Intramuscular fat (IMF) highly relates to meat quality attributes and is a major driver for consumer demand for lamb meat (1, 7–11). The levels of IMF as well as its fatty acid profile contribute to eating quality; an IMF content of 3–5% positively influences the lightness, yellowness, tenderness, flavor and consumer acceptance of beef and lamb (7, 11, 12). In addition, lipids in IMF contribute to meat aroma characteristics, which are related to specific volatile compounds (13–17). Some degradation compounds of *n*-6 unsaturated fatty acids, such as hexanal (*E*,*E*)-2,4-decadienal and 1-octen-3-ol, are important contributors to meat-like flavor notes (18, 19). Previous studies have reported large variation in IMF characteristics within breeds (20, 21). In a previous report, we showed that fatty acid content and the lipid species profile in IMF varied between individual Hu sheep and this variation would influence concentrations of odor-active volatile compounds such as 1-octen-3-ol, 2-pentylfuran, as well as (*E*,*E*)-2,4-decadienal in lamb meat (17). Additionally, Zhang et al. (22) found that the IMF variation existed within Tan sheep and were associated with changes in amnio acids and fatty acid profiles. However, studies investigating differences in volatile compound profiles between Hu and Tan sheep have not meaningfully addressed the normal variation in IMF *within* each breed. Therefore, comparison of meat quality traits, IMF content, fatty acids in IMF, and volatile compounds, between Hu and Tan sheep, is important to improve knowledge of this research area.

The present work aimed to (i) characterize IMF content in psoas major muscle (short loin) from the Hu and Tan experimental groups; (ii) evaluate the influence of breed on physicochemical properties, the fatty acid composition of the IMF and amino acid profiles; and (iii) identify and differentiate key odor-active volatile compounds between Hu and Tan sheep with the representative IMF content of each breed.

## 2. Materials and methods

### 2.1. Reagents and chemicals

2-Ethylfuran, methyl butanoate, 2-methylthiophene, methyl hexanoate, 3-octen-2-one, 2-pentylthiophene, dimethyl sulfone, methanethiol, ethyl acetate, hexanal, acetoin, nonanal, (*E*)-2-nonenal, (*Z*)-5-octen-1-ol, (*E*,*E*)-2,4-decadienal, dimethyl trisulfide, 1-octen-3-one, 1-octen-3-ol, 2-pentylpyridine, 2-acetyl-2-thiazoline, butanoic acid, methyl ester, and 2-methylthiophene were supplied by Aladdin Biochemical Technology (Shanghai, China) as analytical-grade standards. The analytical standards *n*-alkane mixture (C_7_–C_40_), 4-heptanone, 2-heptanone, 3-octanone, 2-octanone, γ-nonalactone, heptanal, octanal and pentanal were from Sigma-Aldrich (Shanghai, China). The gas chromatography standards 2-butylfuran, 2-heptylfuran and 2-pentylfuran were supplied by Alfa Aesar (Shanghai, China). Seventeen amino acids reference standards including glycine, L-alanine, L-serine, L-proline, L-valine, L-threonine, L-leucine, isoleucine, L-aspartic acid, L-lysine, L-glutamic acid, L-methionine, L-histidine, L-phenylalanine, L-arginine, L-tyrosine and L-cystine, each with purity ≥98%, were purchased from Alta Scientific Ltd. (Tianjin, China).

### 2.2. Animals and sampling

All animal procedures were conducted according to the regulations of the Animal Care and Use Committee of the Institute of Animal Sciences of the Chinese Academy of Agricultural Sciences (No. IAS 2020-69). In brief, flocks of 176 Hu male sheep and 76 Tan male sheep, with similar weights, were reared in the same environment, in individually ventilated pens (0.8 × 1.0 m^2^, one sheep per pen). After weaning at 56 days old, all animals were fed *ad libitum* on total mixed ration pellets (Supplementary Table S1) and given free access to water until 6 months old. After fasting for 12 h, all animals were electrically stunned for 3 s (SQ05A stunner, Wujiang Aneng Electronic Technology, Suzhou, China), exsanguinated, eviscerated, and split. Psoas major muscles between the right 1st and 4th lumbar vertebrae were collected at 45 min postmortem for IMF, fatty acid, and amino acid analysis. The remaining psoas major muscles of each left and right carcass were vacuum-packed, and chilled for 24 h (2–4°C) for later determination of meat quality characteristics and volatile analysis. Following analysis of the IMF content, 40 individuals, 20 replicates per group, were selected according to the IMF content in order to reduce the impact of IMF variation within breed on characteristics differences between breeds.

### 2.3. Meat quality characteristics

Muscle pH was recorded with a pH meter (HI99163, Hanna Instruments Inc., Washington, DC, United States). Calibration of the pH probe was performed using pH 4.0 and 7.0 standard buffers. Color coordinates (lightness, L*; redness, a*; yellowness, b*; hue angle; chroma) were determined at three random locations using a Minolta CR-400 colorimeter, equipped with a D65 illuminator, 8 mm aperture and 2° viewing angle (Konica Minolta Sensing Inc., Osaka, Japan). Chroma ($\sqrt{{\left(a^{*}\right)}^{2}+{\left(b^{*}\right)}^{2}}$) and hue angle ($\tan^{-1}\frac{a^{*}}{b^{*}}$) values were calculated according to Honikel (23). Pressing loss was calculated as the difference in weight before and after pressing, divided by initial weight, as reported previously (24). Briefly, a muscle core sample (25 mm diameter, 10 mm thickness) was weighed, pressed for 5 min under a force of 343 N using a dilatometer, then reweighed. Drip loss and cooking loss of psoas major muscles were measured as reported previously (25). For cooking loss determination, samples were weighed, sealed in bags, heated to center temperature of 70°C in a water bath (80°C), cooled, refrigerated, wiped dry, and reweighed. After determining cooking loss, the Warner-Bratzler shear force was conducted, on the same samples, with 10 strips (10 mm × 10 mm × 20 mm) using an HDP/BSW V-shaped blade attached to a texture analyzer (TA.XT. Plus, Stable Micro Systems, Godalming, United Kingdom).

### 2.4. IMF content and fatty acid analysis

Intramuscular fat was extracted using the Soxhlet extraction method reported previously (25) and indicated as g/100 g wet muscle sample. After saponification, fatty acids from muscle tissues were reacted with sodium methoxide to produce fatty acid methyl esters and methyl undecanoate was employed as internal standards. The obtained methyl esters were quantitated using an Agilent 6890 gas chromatograph with DB-23 capillary column (60 m × 0.25 mm i.d., 0.25 μm film thickness) coupled with a flame-ionization detector (Agilent Technologies, Santa Clara, CA, United States). Helium as carrier gas was set at a flow rate of 3.0 ml/min with a split ratio of 1 to 20. The temperature gradient was as follows: 100–220°C with increasing rate at 4°C/min, followed by 5°C/min to 250°C, which was maintained for another 5 min. The temperature of inlet was set at 250°C while that of flame-ionization temperature was shown at 280°C. Fatty acid methyl esters were identified using retention time, confirmed by authentic standards, and quantitated using internal standard based method (methyl undecanoate).

### 2.5. Total and free amino acid analysis

For total amino acid analysis, psoas major muscles (~1 g) were hydrolyzed with HCl (10 ml, 6 mol/l) at 110°C for 22 h. Hydrolysates were transferred, filtered, and diluted with deionized water to volume. The filters were dried under nitrogen and the residue redissolved in HCl aqueous solution (2 ml, 0.02 mol/l) for further analysis. Amino acid concentrations were determined on an L-8900 amino acid analyzer (Hitachi, Tokyo, Japan). Identification of amino acids was performed by comparison with authentic standards.

For free amnio acid analysis, psoas major muscles (~100 mg) and 1 ml of sulphosalicylic acid (30 g/l) were mixed, homogenized, and centrifuged for 15 min at 14000 rpm at 4°C. The supernatant was vortex-mixed with hexane, centrifuged, and filtered through a 0.22 μm membrane. The filtrate was carried out an ACQUITY ultra-performance liquid chromatography (UPLC, Waters Corp., Milford, MA, United States) coupled with triple-quadrupole mass spectrometer (SCIEX QTRAP 6500, Framingham, MA, United States) in the electron spray ionization (ESI) mode. Two microliters of the samples were injected into LC–MS/MS system using an autosampler kept at 10°C. An ACQUITY UPLC HSS T3 column (2.1 × 100 mm, particle size 1.8 μm, pore size 100 Å) was applied to separate target analysts in meat samples. Mobile phase A was deionized water while mobile phase B was methanol. The 10-min gradient conditions were set as follows: 0–3 min, 2% B; 3–7 min, 2–95% B; 7–8 min, 95% B; 8–8.1 min, 95–2% B; 8.1–10 min, 2% B. The MS parameters was an ion-spray voltage of 4500 V, turbo gun source temperature of 400°C and curtain gas of 300 psi. Free amino acids were quantitated by external standard curves containing varying concentrations of reference standards.

### 2.6. Volatile compound analysis

Short loins were cut into small pieces (30 mm × 25 mm × 15 mm), sealed in bags, vacuum packed, heated for 60 min in an 80°C-water bath, cooled, snap-frozen, and ground. Cooked meat samples (~4.0 g) were weighed into a 20 ml vial and 10 μl of 2-methyl-3-heptanone (0.05 μg/l in methanol) were added as an internal standard. The vial was incubated for 20 min at 55°C. A solid-phase microextraction fiber coated with a 50/30 divinylbenzene/Carboxen^®^/polydimethylbenzene fiber (Supelco, Bellefonte, PA, United States) was exposed to the headspace for 40 min to extract the volatile compounds. After extraction, the fiber was injected to desorb for 3 min at 250°C in split mode, and the split injection were set with a split ratio of 5:1.

Volatile compounds were separated with a VF-WAXms capillary column (60 m × 0.25 mm i.d., 0.25 μm film thickness, Agilent Technologies, Santa Clara, CA, United States) attached to Q Exactive GC/Orbitrap-MS (Thermo Fisher Scientific, Waltham, MA, United States). Chromatographic conditions and identification of volatile compounds were carried out as previously reported (17). Volatile compounds were first semi-quantified by an internal standard method. Then compounds having odor activity values (OAVs) greater than 1 were accurately quantified using external standard curves, constructed with various concentrations of standards (10 ‍mg/L–10 ‍g/L) in an odorless matrix. After mixing with the odorless matrix, the reference compounds were extracted and determined under the same analysis conditions. The odorless matrix was prepared as described previously (26), and was mainly composed of benzene, ethylbenzene, o-xylene, styrene, and linear alkane hydrocarbons such as octane, heptane, and nonane. The OAVs were calculated as dividing each compound concentration by its reported odor threshold in water (27).

### 2.7. Data analysis

Data were expressed as the mean ± standard error of the mean. Statistical analyses were performed using the SPSS software (Version 22.0, SPSS, Inc., Chicago, IL, United States). The significance of the differences in meat quality parameters, fatty acid profiles in IMF, amino acid profiles, and odor-active volatile compounds were determined by multivariate analysis of variance using breed as a fixed factor and IMF as a covariate. Pearson’s correlation coefficients were conducted with meat quality characteristics and the IMF, and a two-sided test for significance was performed. The significant differences were identified as *p* < 0.05.

## 3. Results

### 3.1. Comparison of IMF and meat quality attributes between Hu and Tan sheep

Large variations in intra-breed IMF content were found in psoas major muscles from Hu (1.38–6.93%) and Tan (1.83–6.39%) sheep, but no inter-breed difference in IMF content was observed (*p* > 0.05; Table 1). The mean IMF content of the selected samples was 3.27% ± 0.03% for Hu sheep and 3.18% ± 0.05% for Tan sheep, thereby more accurately representing the IMF average of each breed and better reflecting meat quality differences between breeds. The IMF content and meat quality characteristics in selected samples were shown in Table 2. There was no inter-breed difference in IMF, pH, or pressing loss (*p* > 0.05; Table 2). Drip loss was 32.5% lower for Tan sheep (*p* < 0.01; Table 2). Cooking loss and shear force were 8.2 and 28.5% higher, respectively for Tan sheep (*p* < 0.01; Table 2). Higher lightness, redness, yellowness, and chroma value, and a lower hue angle were observed in Tan sheep, indicating that muscles from Tan sheep had a more intense red color (*p* < 0.01; Table 2). Meat quality characteristics did not have an obvious correlation with the IMF content (Table 2).

**Table 1:** Descriptive statistics of intramuscular fat content (IMF; % *w*/*w*) in Hu and Tan sheep.

Item	Hu sheep (*N* = 176)	Tan sheep (*N* = 76)
Mean	3.41	3.35
Standard deviation	1.03	0.95
Standard error of mean	0.08	0.11
Median	3.27	3.25
Range	1.38–6.93	1.83–6.39
Variance	1.06	0.91
Value of *p*	0.670	

**Table 2:** Meat quality characteristics and intramuscular fat of psoas major muscles from Hu and Tan sheep (*n* = 20).

Item	Hu sheep	Tan sheep	*p*_breed_	*r*_IMF_	*p*_IMF_
pH	6.18 ± 0.08	5.97 ± 0.07	0.095	0.053	0.743
Drip loss (%)	2.67 ± 0.22	1.80 ± 0.10	0.008	0.173	0.314
Pressing loss (%)	27.10 ± 0.87	26.54 ± 0.81	0.687	0.144	0.387
Shear force (N)	64.18 ± 3.78	82.48 ± 2.32	0.001	−0.140	0.403
Cooking loss (%)	37.58 ± 0.79	40.86 ± 0.84	0.003	−0.238	0.145
L*	34.58 ± 0.54	37.41 ± 0.51	0.003	−0.014	0.936
a*	19.47 ± 0.35	20.74 ± 0.18	0.007	−0.046	0.792
b*	5.79 ± 0.38	7.71 ± 0.28	0.001	−0.035	0.840
Hue angle	73.65 ± 0.76	69.71 ± 0.56	<0.001	0.036	0.837
Chroma	20.34 ± 0.44	22.15 ± 0.25	0.002	−0.043	0.803
Intramuscular fat content (%)	3.27 ± 0.03	3.18 ± 0.05	0.127		

*p*_breed_ = significance for breed comparison, *r*_IMF_ = correlation coefficient between meat quality traits and intramuscular fat and associated significance (pIMF).

### 3.2. Differences in amino acid and fatty acid composition between Hu and Tan sheep

**Figure 1:**
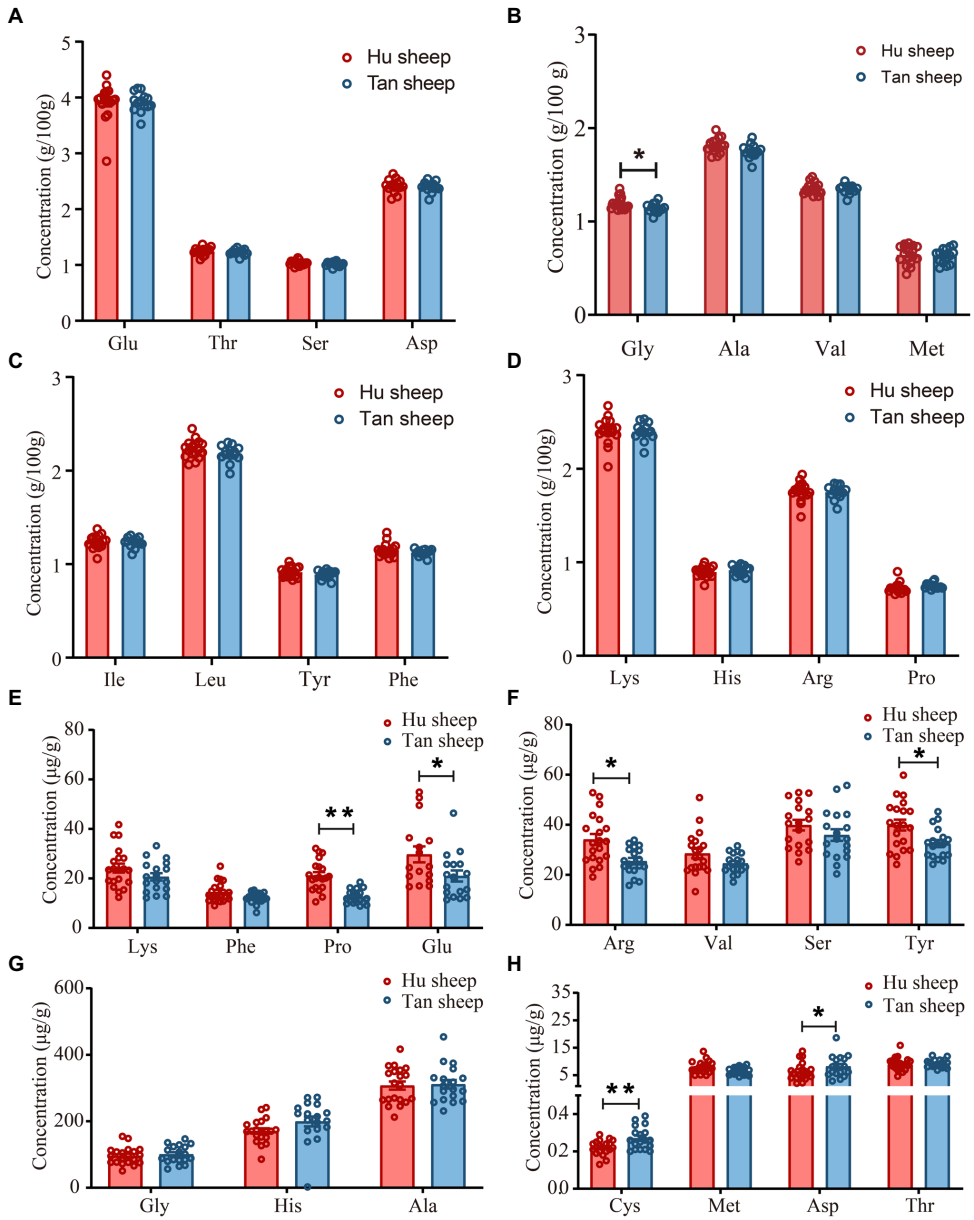
**(A–D)** Total and **(E–H)** free amino acid profiles of psoas major muscles from Hu and Tan sheep (*n* = 20). Gly, glycine; Ala, L-alanine; Val, L-valine; Leu, L-leucine; Ile, isoleucine; Pro, L-proline; Phe, L-phenylalanine; Tyr, L-tyrosine; Ser, L-serine; Thr, L-threonine; Met, L-methionine; Asp., L-aspartic acid; Glu, L-glutamic acid; Lys, L-lysine; Arg, L-arginine; His, L-histidine; Cys, L-cystine. ^*^p < 0.05, ^**^p < 0.01.

**Figure 2:**
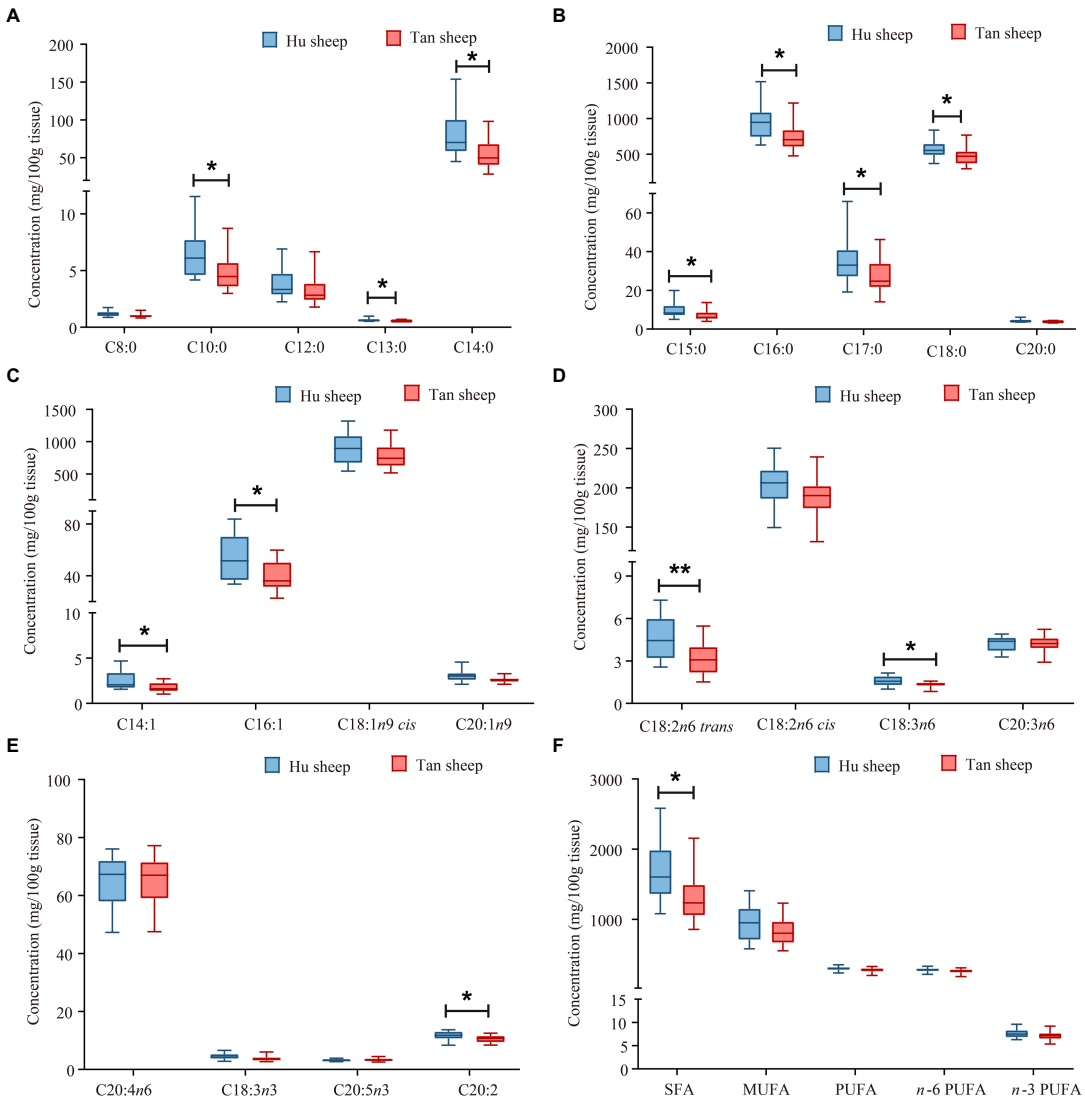
Fatty acid profiles of psoas major muscles from Hu and Tan sheep (*n* = 20). C8:0, caprylic acid; C10:0, capric acid; C12:0, lauric acid; C13:0, tridecanoic acid; C14:0, myristic acid; C15:0, pentadecanoic acid; C16:0, hexadacanoic acid; C17:0, heptadecanoic acid; C18:0, octadecanoic acid; C20:0, arachidic acid; C14:1, myristoleic acid; C16:1, palmitoleic acid; C18:1*n*9 cis, oleic acid; C20:1*n*9 cis-11-eicosenoic acid; C18:2*n*6 trans, linolelaidic acid; C18:2*n*6 cis, linoleic acid; C18:3*n*6, γ-linolenic acid; C20:3*n*6, cis-8,11,14-eicosatrienoic acid; C20:4*n*6, arachidonic acid; C18:3*n*3, α-linolenic acid; C20:5*n*3, cis-5,8,11,14,17-eicosapentaenoic acid; C20:2, cis-11,14-eicosadienoic acid; SFA, total saturated fatty acids; MUFA, total monounsaturated fatty acids; PUFA, total polyunsaturated fatty acids, *n*-6 PUFA, total omega-6 polyunsaturated fatty acids; *n*-3 PUFA, total omega-3 polyunsaturated fatty acids. ^*^p < 0.05 and ^**^p < 0.01.

Total glycine concentration was lower in Tan sheep compared to Hu sheep (*p* < 0.05; Figure 1). Free L-proline, L-arginine, L-tyrosine, and L-glutamic acid were 18.5–40.3% lower in Tan sheep against Hu sheep (*p* < 0.05; Figures 1E,F). Free L-cystine and L-aspartic acid were 22.4% and 13.6% higher, respectively in Tan sheep relative to Hu sheep (*p* < 0.05; Figure 1H). Tan sheep muscle was 378 mg/100 g lower (*p* < 0.05) in total saturated fatty acids, reflecting lower concentrations of C18:0, C16:0, and C14:0 than Hu sheep (*p* < 0.05; Figures 2A,B,F). Concentrations of C16:1, C18:2*n*6 *trans*, C18:3*n*6, and C20:2 were lower in Tan sheep muscle (*p* < 0.05; Figures 2C–E). No differences were detected for contents of C18:1*n*9 *cis* and C20:1*n*9 (*p* > 0.05; Figure 2C).

### 3.3. Comparison of volatile compound profiles between Hu and Tan sheep

**Figure 3:**
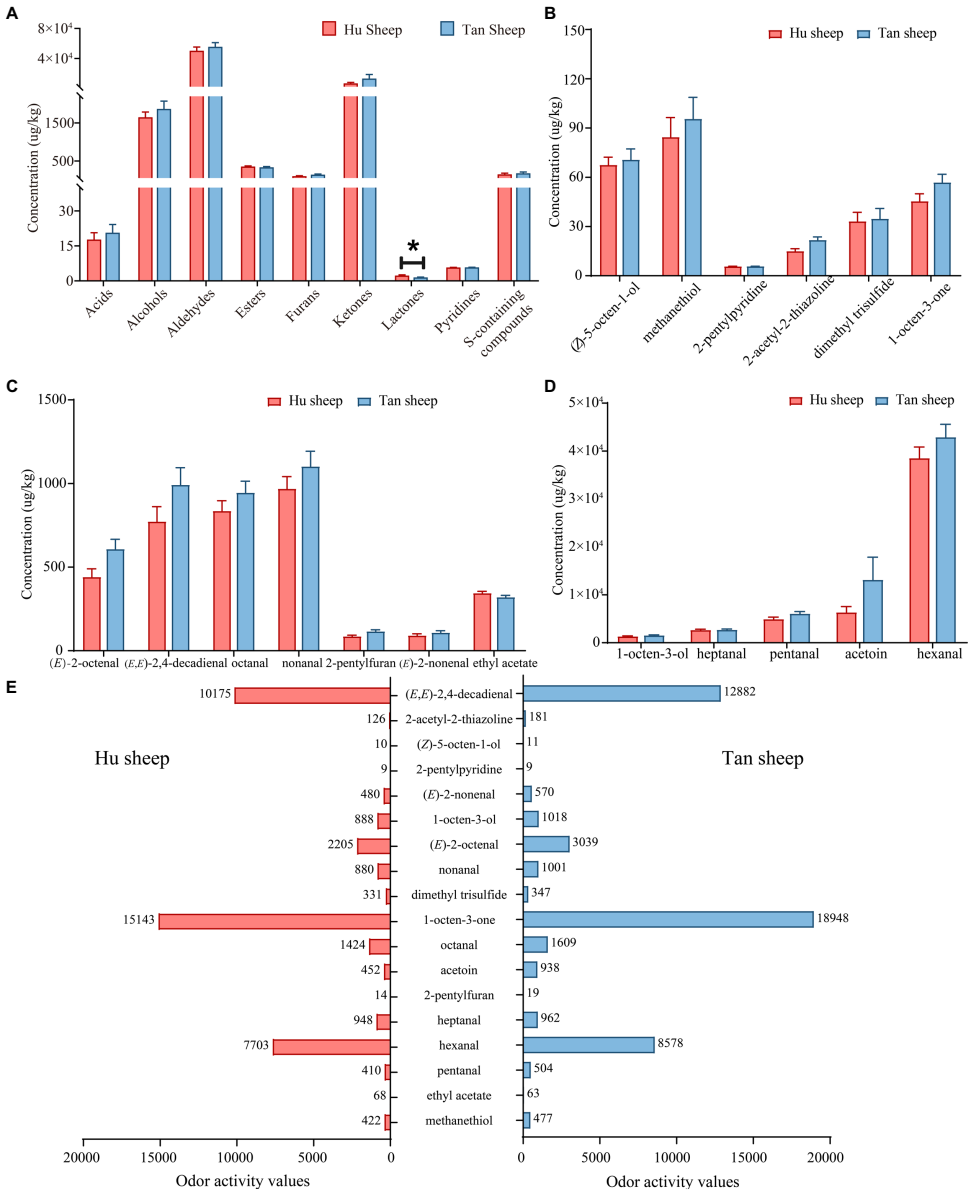
Comparative concentrations of **(A)** volatile compound classes; **(B–D)** the odor-active volatile compounds with odor activity values > 1; **(E)** Odor activity values of 18 odor-active volatile compounds in Hu and Tan sheep (*n* = 20). ^*^p < 0.05.

Fifty-three volatile compounds were identified and quantified in Hu and Tan sheep, namely, 14 aldehydes, nine ketones, eight esters, seven S-containing compounds, six alcohols, four furans, three acids, one lactone and one pyridine (Table 3). The total concentrations of nine chemical classes were quantified for inter-breed comparison (Figure 3); the concentration of γ-nonalactone was significantly decreased in psoas major muscles from Tan sheep against Hu sheep (*p* < 0.05), but no other differences between breeds were observed (*p* > 0.05; Figure 3A).

**Table 3:** Volatile compounds identified in Hu and Tan sheep (*n* = 20).

NO.	Compound^a^	Linear retention index		Identification^d^
		Calculated^b^	Literature^c^	
1	Octanoic acid	2,063	2,077	MS, LRI, Std
2	Nonanoic acid	2,170	2,171	MS, LRI
3	Decanoic acid	2,276	2,289	MS, LRI, Std
4	Pentanol	1,247	1,253	MS, LRI, Std
5	1-Octen-3-ol	1,448	1,451	MS, LRI, Std
6	Octanol	1,556	1,557	MS, LRI
7	(*Z*)-5-Octen-1-ol	1,615	1,619	MS, LRI, Std
8	Nonanol	1,658	1,660	MS, LRI
9	Benzenemethanol	1,883	1,890	MS, LRI, Std
10	Propanal	797	801	MS, LRI, Std
11	Pentanal	982	991	MS, LRI, Std
12	Hexanal	1,085	1,089	MS, LRI, Std
13	Heptanal	1,190	1,192	MS, LRI, Std
14	(*E*)-2-Hexenal	1,225	1,230	MS, LRI, Std
15	Octanal	1,295	1,298	MS, LRI, Std
16	(*E*)-2-Heptenal	1,331	1,335	MS, LRI, Std
17	Nonanal	1,400	1,402	MS, LRI, Std
18	(*E*)-2-Octenal	1,438	1,440	MS, LRI, Std
19	(*E*)-2-Nonenal	1,545	1,547	MS, LRI, Std
20	(*E*)-2-Decenal	1,654	1,644	MS, LRI
21	4-Ethyl benzaldehyde	1,724	1,721	MS, LRI
22	2-Undecenal	1,762	1,751	MS, LRI
23	(*E*,*E*)-2,4-Decadienal	1,822	1,823	MS, LRI, Std
24	Ethyl acetate	890	896	MS, LRI, Std
25	Propyl acetate	976	973	MS, LRI
26	Methyl butanoate	989	994	MS, LRI, Std
27	Methyl hexanoate	1,190	1,196	MS, LRI, Std
28	Octyl formate	1,433	1,437	MS, LRI, Std
29	Ethyl octanoate	1,439	1,440	MS, LRI, Std
30	Methyl decanoate	1,600	1,593	MS, LRI
31	Ethyl decanoate	1,643	1,644	MS, LRI, Std
32	2-Ethylfuran	955	960	MS, LRI, Std
33	2-Butylfuran	1,137	1,142	MS, LRI, Std
34	2-Pentylfuran	1,237	1,241	MS, LRI, Std
35	2-Heptylfuran	1,440	1,444	MS, LRI, Std
36	2-Butanone	905	908	MS, LRI, Std
37	2-Pentanone	980	981	MS, LRI
38	4-Heptanone	1,129	1,133	MS, LRI, Std
39	2-Heptanone	1,186	1,190	MS, LRI, Std
40	3-Octanone	1,259	1,264	MS, LRI, Std
41	2-Octanone	1,290	1,293	MS, LRI, Std
42	Acetoin	1,290	1,298	MS, LRI, Std
43	1-Octen-3-one	1,307	1,310	MS, LRI, Std
44	3-Octen-2-one	1,414	1,417	MS, LRI, Std
45	γ-Nonalactone	2,046	2,051	MS, LRI, Std
46	2-Pentylpyridine	1,580	1,584	MS, LRI, Std
47	2-Methylthiophene	1,098	1,104	MS, LRI, Std
48	Dimethyl trisulfide	1,394	1,398	MS, LRI, Std
49	2-Pentyl-thiophene	1,469	1,473	MS, LRI, Std
50	2-Acetyl-2-thiazoline	1,773	1,778	MS, LRI, Std
51	Dimethyl sulfone	1,913	1,920	MS, LRI, Std
52	Benzothiazole	1,976	1,981	MS, LRI, Std
53	Methanethiol	674	675	MS, LRI, Std

a Volatile compounds identified by HS-SPME-GC–MS.

b Calculated data based on a series of n-alkanes (C_7_–C_40_).

c Reported data obtained from the online database: http://www.flavornet.org/; https://pubchem.ncbi.nlm.nih.gov./.

d MS, identified using MS spectra; LRI, identified using the linear retention index; Std, confirmed by comparisons with authentic standards.

To assess volatile compounds’ contribution to the overall aroma, their concentrations and OAVs were compared. Eighteen compounds having OAVs greater than 1 were accurately quantitated using an external standard method. Table 4 lists the odorants, the selected ion fragments, calibration equations, and correlation coefficients. Good linearity of detector response for the odor-active volatile compounds was observed (*R*^2^ > 0.99; Table 4). The major compounds in Tan sheep were hexanal at 42.89 mg/kg, followed by acetoin (13.14 mg/kg), pentanal (6055.35 μg/kg), nonanal (1102.16 μg/kg), heptanal (2697.63 μg/kg), (*E*,*E*)-2,4-decadienal (991.98 μg/kg), and 1-octen-3-ol (1527.90 μg/kg; Figure 3). Similarly, high levels of hexanal (38.52 mg/kg), acetoin (6339.21 μg/kg), 1-octen-3-ol (1333.58 μg/kg), pentanal (4931.36 μg/kg), and heptanal (2656.19 μg/kg) were found in Hu sheep. No differences between breeds were observed for hexanal, pentanal, (*E*,*E*)-2,4-decadienal, heptanal, acetoin, 1-octen-3-ol, nonanal, octanal, 2-pentylfuran, (*E*)-2-nonenal, ethyl acetate, 2-pentylpyridine, dimethyl trisulfide, methanethiol, 1-octen-3-one, (*Z*)-5-octen-1-ol, (*E*)-2-octenal, and 2-acetyl-2-thiazoline (*p* > 0.05; Figures 3B–D).

**Table 4:** Ions used for quantitation, calibration equations, coefficients of determination (*R*^2^), and odor threshold of 18 odorants in Hu and Tan sheep.

Compounds	Ion (*m*/*z*)^a^	Calibration equations^b^	*R* ^2^	Odor threshold (μg/kg)^c^	Odor description^d^
Dimethyl trisulfide	125.9626, 110.9391, 78.9670, 61.0106	*y* = 110.3*x*–2.1703	0.9901	0.1	sulfur, meaty
2-Pentylpyridine	93.0573, 106.0651, 120.0807, 78.0338	*y* = 0.0733*x* + 0.0427	0.9901	0.6	fat
Pentanal	44.0256, 43.0178, 41.0385, 58.0413	*y* = 16.106*x*–0.1433	0.9998	12	almond, malt, pungent
Octanal	81.0698, 69.0698, 67.05423, 95.0855	*y* = 4.1648*x*–0.6054	0.9972	0.587	fat, soap, lemon, green
Nonanal	81.0698, 95.0855, 67.0542, 70.0777	*y* = 1.1475*x*–0.5377	0.9960	1.1	fat, citrus, green
Methanethiol	46.9950, 44.9793, 48.0028, 45.9871	*y* = 11.462*x*–0.0318	0.9904	0.2	sulfur, sweat
Hexanal	67.0542, 72.0569, 44.0256, 57.0334, 82.0777	*y* = 26.723*x*–4.0711	0.9913	5	grass, tallow, fat
Heptanal	81.0698, 70.0777, 44.0256, 55.0542, 86.0726	*y* = 8.1759*x*–0.345	0.9987	2.8	fat, citrus, rancid
2-Pentylfuran	81.0334, 94.0777, 138.1039, 53.0385	*y* = 0.1204*x* + 0.0103	0.9978	5.8	vegetable, earthy
Acetoin	45.0334, 42.0100, 43.0178, 88.0518	*y* = 158.49*x* + 23.804	0.9910	14	butter, cream
Ethyl acetate	43.0178, 61.0284, 45.0334, 70.0413	*y* = 6.7343*x* + 0.2185	0.9901	5	pineapple
(*E*)-2-Nonenal	83.0491, 55.0542, 93.0698, 111.0804	*y* = 8.4754*x*–0.0614	0.9937	0.19	cucumber, fat, green
2-Acetyl-2-thiazoline	129.0242, 43.0178, 101.0293, 60.0028	*y* = 5.066*x*–0.0263	0.9968	0.12	roast, pop-corn
(*E*,*E*)-2,4-Decadienal	81.0334, 67.0542, 95.0491, 123.0804	*y* = 46.886*x*–0.242	0.9908	0.077	fried, fat
(*E*)-2-Octenal	83.0491, 55.0542, 67.0542, 93.0698	*y* = 28.244*x*–0.2761	0.9923	0.2	grilled meat, peanut cake
1-Octen-3-one	97.0647, 55.0178, 70.0413, 83.0491	*y* = 24.389*x*–0.0397	0.9983	0.003	mushroom, metal
1-Octen-3-ol	57.0334, 81.0698, 85.0647, 99.0804	*y* = 2.8062*x*–0.6046	0.9971	1.5	mushroom
(*Z*)-5-Octen-1-ol	67.0542, 79.0542, 81.0698, 95.0855	*y* = 2.7405*x*–0.019	0.9985	6	oil

a Selected ions used in quantitative analysis.

b Variables: *x* is the peak area relative to that of the internal standard, 2-methyl-3-heptanone, and *y* is the concentration in the sample relative to that of the internal standard, 2-methyl-3-heptanone.

c Odor description was taken from online database (http://www.flavornet.org/; http://www.odour.org.uk./).

d Odor threshold in water obtained from the literature (27).

As presented in Figure 3E, the concentrations of 18 odor-active volatile compounds were greater than their odor thresholds (OAV > 1) in Hu and Tan sheep, indicating that these compounds make important contributions to the aroma profile of lamb meat. The compound with the highest OAV in Tan sheep was 1-octen-3-one, followed by hexanal, octanal, (*E*)-2-octenal, and (*E*,*E*)-2,4-decadienal with OAVs > 1,000, whereas 2-pentylpyridine, 2-pentylfuran, ethyl acetate, and (*Z*)-5-octen-1-ol had low OAVs between 9 and 19. In addition, no significant differences were observed between Hu and Tan sheep in the 18 odor-active volatile compounds. Taking all together, the odor-active volatile compound profiles of meat from Hu sheep and Tan sheep with the 3–3.3% IMF differed only slightly.

## 4. Discussion

There was a much larger variation within breeds, in IMF content. Variation in IMF within breeds affects meat quality characteristics such as shear force, texture, and color coordinates (1, 22). Consumer sensory evaluation shows that lamb overall palatability increases markedly at around 3% IMF and peaks at 4% IMF (7). The selected samples with 3–3.3% IMF, as done here, were conducted to study differences in characteristics between breeds, with adjustment for IMF differences using IMF as a covariate.

The IMF content makes a considerable contribution to some meat quality traits such as shear force, color coordinates, and flavor (12, 28). It has been shown in pork from high-fat Duroc pigs that the IMF content linearly related to the Warner-Bratzler shear force (29). Zhang et al. concluded that the IMF content positively correlated with color coordinates, namely, lightness, redness, yellowness, and the IMF variation within Tan sheep was ascribed to the difference in meat color (22). Inconsistent with these findings, the IMF content did not correlate with shear force or color coordinates in the present study. The IMF content in psoas major muscles did not differ between Hu and Tan sheep, in agreement with a previous report (6). Other meat quality traits were different between breed, except for pH and pressing loss. Those findings were in line with a previous report, that no difference was observed in the ultimate pH, whereas color and cooking loss were significantly different (30). Muscle fat from Hu and Tan sheep contained relatively large amounts of C18:1*n*9 *cis*, C18:2*n*6 *cis*, C16:0, and C18:0 fatty acids, accounting for 89.94% of the total, consistent with previous studies in which these four fatty acids were the predominant fatty acids in lamb (31, 32). Of these four major fatty acids, the levels of C18:1*n*9 *cis* and C18:2*n*6 *cis* were no significant difference between Hu and Tan sheep, whereas that of C16:0 and C18:0 significantly decreased in the latter breed. No significant difference was detected in the concentrations of total monounsaturated fatty acids, *n*-3 and *n*-6 polyunsaturated fatty acids, and total polyunsaturated fatty acids, in both breeds. Saturated fatty acids are partially oxidized during cooking to hydrocarbons or alcohols, but their contributions to meat aroma are minimal, because of high odor thresholds. However, unsaturated fatty acids are the major precursors of the lipid-derived aldehydes, ketones, and furans that mainly contribute to meat aroma (33). Thus, the fatty acid compositional differences between Hu and Tan sheep may make a minor influence to the lipid-derived volatile compound profile.

Comparison of concentrations and OAVs identified 18 odorants which make sizable contributions to the aroma profile of lamb meat. Notably, eight aldehydes were identified, which made up approximately half of the odorants. (*E*)-2-Octenal, (*E*)-2-nonenal, (*E*,*E*)-2,4-decadienal, hexanal, octanal, heptanal, and nonanal were the predominant aldehydes due to their high contents and OAVs in Hu and Tan sheep. This agrees with previous reports that these seven odorants are the dominant contributors to the overall aroma of lamb (17, 34, 35). These aldehydes can be generated from thermal oxidation and decomposition of lipids (19, 33). Hexanal provides the “green grassy” note of stewed mutton and mainly originates from *n*-6 polyunsaturated fatty acids (34). Octanal and nonanal have “fruity” notes and (*E*,*E*)-2,4-decadienal contributes to the “biscuit/fatty/scallion/toasted” aroma of cooked lamb (34). These three aldehydes can be produced through the thermal oxidation of *n*-3 polyunsaturated fatty acids such as linoleic acid as well as docosahexaenoic acid (36). (*E*)-2-Octenal contributes to the “grilled meat” and “peanut cake” notes of meaty flavors and can be derived from linoleic acid (34). In addition, 2-pentylfuran, 1-octen-3-ol, and 1-octen-3-one are also generated by fatty acid oxidation during cooking. Linoleic acid and arachidonic acid are oxidized to 1-octen-3-ol and 1-octen-3-one during heating (37). The *n*-3 or *n*-6 polyunsaturated fatty acids, such as linoleic acid as well as linolenic acid, can undergo thermal oxidation to form 2-pentylfuran (17). 1-Octen-3-one and 1-octen-3-ol confer a “mushroom-like” note, and 2-pentylfuran confers a “vegetable/earthy” note, and all three are important contributors to the overall lamb flavor (34, 38).

The Milliard reaction products, 2-acetyl-2-thiazoline, 2-pentylpyridine, methanethiol, and dimethyl trisulfide, were also found to be odor-active volatile compounds, consistent with previous reports (34, 35), in which they were important contributors to “meaty” aroma. The Maillard reaction of valine and linoleic acid produces 2-pentylpyridine, contributing a “fatty” odor (39, 40). The sulfur amino acid, S-methyl-L-cysteine, and its sulfoxide can undergo thermal oxidation to form dimethyl trisulfide, which contributes to “meaty” and “sulfur” aromas (38, 41). The Strecker degradation of methionine produces methanethiol, which has “sulfur” and “sweat” notes (34, 42). 2-Acetyl-2-thiazoline confers “pop-corn” and “meaty” notes (43). Additionally, it has been reported that γ-nonalactone was an important contributor to the sweet flavor of Wagyu (44) and may originate from the lactonization of hydroxy fatty acids (42). The concentration of γ-nonalactone was higher in Hu sheep against Tan sheep. Overall, the odor-active volatile compounds (*E*)-2-nonenal, (*E*)-2-octenal, octanal, nonanal, (*E*,*E*)-2,4-decadienal, hexanal, heptanal, 1-octen-3-one, 1-octen-3-ol, 2-pentyfuran, methanethiol, dimethyl trisulfide, 2-acetyl-2-thiazoline, 2-pentylpyridine, and γ-nonalactone were the dominant contributors to the lamb meat odor profile.

The concentrations of odor-active volatile compounds were generally similar between Hu and Tan sheep, indicating that the overall meat aromas of the two breeds are similar. Similarly, there was no difference in aroma characteristics between the Hampshire Down, Suffolk, and Santa Inês sheep breeds (45). However, Zhang et al. found clear differences in volatile compound profiles between Hu, Tan, and Dorper sheep, using principal component analysis (6).

Minor concentration differences in odorants between Hu and Tan sheep in this study may result from differences in fatty acid composition. As discussed above, 11 of the 18 major odorants can be derived from unsaturated fatty acids by heating/oxidation. Although some fatty acids, such as C18:2*n*6 *trans*, C20:2, as well as C18:3*n*6 differed significantly, there were no significant differences in the contents of total mono-and poly-unsaturated fatty acids, or the predominant fatty acids C18:2 *n*6 *cis* and C18:1*n*9 *cis*. Therefore, the aroma profiles of Hu and Tan sheep with around 3–3.3% IMF, differed only slightly.

## 5. Conclusion

This study investigated differences in meat quality traits, fatty acids in IMF, amino acids, and volatile compounds *between* Hu and Tan sheep, taking into account the normal IMF variation *within* breeds. There was considerable intra-breed variation in IMF in both Hu and Tan breeds, but the inter-breed IMF content was very similar. Tan sheep meat had lower drip loss, but higher shear force, lightness, redness, and chroma value than Hu sheep. Free L-cystine and L-aspartic acid concentrations in Tan sheep were 1.22 times and 1.13 times that of Hu sheep, respectively. Contents of C16:0 and C18:0 saturated fatty acids were lower in Tan sheep IMF, but concentrations of C18:1*n*9 *cis*, C18:2*n*6 *cis*, total mono-and poly-unsaturated fatty acids were similar to Hu sheep IMF. Volatile compound analysis identified 53 volatile compounds, of which 18 were considered as odor-active volatile compounds (OAV > 1) that mainly contributed to lamb meat aroma. The concentrations of these 18 odor-active volatile compounds in Tan sheep IMF did not significantly differ than Hu sheep IMF, which may result in minor differences in lamb meat odor of Hu and Tan sheep. These findings lay a foundation for further elucidating the reasons for the difference in overall meat quality between Hu and Tan sheep.

## Data availability statement

The raw data supporting the conclusions of this article will be made available by the authors, without undue reservation.

## Ethics statement

The animal study was reviewed and approved by the Animal Care and Use Committee of the Institute of Animal Sciences of the Chinese Academy of Agricultural Sciences.

## Author contributions

JL: writing—original draft, writing—review and editing, investigation, and formal analysis. CT: writing—review and editing, conceptualization. YY: methodology and validation. YH: investigation. QZ: project administration. QM, FL, and JZ: supervision and conceptualization. XY: supervision and resources. All authors contributed to the article and approved the submitted version.

## Funding

The Key Research and Development Plan in Ningxia Hui Autonomous Region (2022BBF02020). the Independent Project of State Key Laboratory of Animal Nutrition (2004DA125184G2205). the Chinese Academy of Agricultural Science and Technology Innovation Project (CAAS-XTCX20190025-8 and ASTIP-IAS-12).

## Conflict of interest

The authors declare that the research was conducted in the absence of any commercial or financial relationships that could be construed as a potential conflict of interest.

## Publisher’s note

All claims expressed in this article are solely those of the authors and do not necessarily represent those of their affiliated organizations, or those of the publisher, the editors and the reviewers. Any product that may be evaluated in this article, or claim that may be made by its manufacturer, is not guaranteed or endorsed by the publisher.
