# Supplementary material for: Characterization of meat quality traits, fatty acids and volatile compounds in Hu and Tan sheep
Source: Front Nutr. 2023 Feb 14;10:1072159. doi: 10.3389/fnut.2023.1072159 (PMC9971989; doi:10.3389/fnut.2023.1072159)
Supplement: Supplementary file 1 [file Table_1.DOCX]

**Table S1.** Composition and nutritional values of experimental diets.

| Items | Diet |
| --- | --- |
| Ingredient (%) | |
| corn | 32.5 |
| corn germ meal | 18 |
| corn stalks | 12 |
| corn hulls | 11.2 |
| corn cob | 8 |
| soybean meal | 5 |
| cotton meal | 5 |
| molasses | 3.3 |
| bentonite | 1.5 |
| baking soda | 1 |
| stone powder | 0.8 |
| expanded urea | 0.5 |
| premix | 0.5 |
| Nutrient level (%) | |
| digestible energy, MJ/kg | 11.11 |
| dry matter | 88.78 |
| crude protein | 13.09 |
| crude fiber | 9.78 |
| acid-detergent fiber | 13.99 |
| neutral-detergent fiber | 27.08 |
